# The self, its body and its brain

**DOI:** 10.1038/s41598-023-39959-w

**Published:** 2023-08-07

**Authors:** Morten Overgaard, Catherine Preston, Jane Aspell

**Affiliations:** 1 CFIN, Department of Clinical Medicine, Aarhus University, Aarhus, Denmark; 2 Department of Psychology, University of York, York, UK; 3 School of Psychology and Sport Science, Anglia Ruskin University, Cambridge, UK

**Keywords:** Neuroscience, Human behaviour

## Abstract

The body is intrinsic to our sense of self and as such, any theoretical account of the self should also include contributions of the body. This Collection incorporates a series of papers that demonstrate the inextricable relationship between body and self. The papers include studies of body illusions and studies of observed differences in bodily experience in participants with psychiatric and physical conditions. Papers in the Collection also address methodological issues, because measuring and manipulating the bodily self does not come without challenges; subjective experiences are difficult to capture empirically. Making progress on these methodological limitations is crucial to further develop experimental design and thus our understanding of self-body relations.

Cognitive neuroscience and its neighbouring disciplines have for decades investigated human behaviour and its neural correlates. By far, most of this research has conceived of mental states as “internal”—states that exist inside the heads of individuals. This approach has led to many important findings, yet has also had to confront important challenges. One such problem is that the cognitive concept of mind has been historically defined and investigated context-free—even excluding the rest of the body, of which the brain is one part. Another problem is that the cognitive concept of mind in most cases only implicitly refers to “the self”—the subject having the mental states in question. Well-known classical cognitive models (e.g. working memory^1^) as well as more recent models (e.g. Prediction Coding^2^) only refer to an experiencing or subjective self implicitly. Indeed, an experiencing subject/self is rarely directly addressed.

However, every feeling, thought and behaviour occurs in the context of the body, and thus it is intuitive that embodied experience may impact our mental processing and sense of self, and vice versa. Examples of this relationship can be found within papers from the current Collection, with the suggestion that the way we process external stimuli is mediated by an interaction between our own expectations and their locations relative to the body^3^. Additionally, many people with psychiatric conditions experience differences in bodily experience. For example, people with schizophrenia often have disrupted body awareness^4^ and people with eating disorders might have impaired multisensory processing^5^ or experience reduced pleasure from touch^6^. Moreover, physical conditions, such as chronic pain and changes in the body that occur as a result of pregnancy and childbirth are also thought to be related to changes in our bodily experience^7,8^.

The concept of “the self” has a peculiar and complicated place in cognitive science. Ever since William James^9^ outlined different concepts of the self, philosophers and psychologists have worked to refine these concepts. Supplementing James' inventory of physical self, mental self, spiritual self, and the ego, Neisser^10^ suggested distinctions between ecological, interpersonal, extended, private, and conceptual aspects of self. More recently, reviewing a contentious multidisciplinary collection of essays, Strawson^11^ found an overabundance of delineations between cognitive, embodied, fictional, and narrative selves, among others.

Whereas some of these definitions fit into classical cognitive science categories—e.g. the self as a meta-representation—the most fundamental concept is the self as “subjectivity” or “a point of view”^12^. In any conscious experience, the experienced object is experienced from a certain point of view (“my own”) and all conscious experiences are experienced by a conscious *experiencer* or subject*.* The existence of a self in this understanding is derivable from any possible experience, as even experiences without self-knowledge are experienced from a specific point of view^13^. According to this logic, an organism that never has any subjective experiences is not considered a “subject”, while an organism that has even the most rudimentary experiences has all that is required to be considered a “subject”. In this way, the concepts of consciousness and self necessitate each other at a very basic level. Taken together, these illustrate some of the challenges in cognitive neuroscience that are fundamental and seemingly inherent to the discipline. To move forward, we must integrate a number of outstanding questions to answer these challenges.

One key question is: how do we measure and manipulate the self? The attempt to “measure” subjective phenomena has unfolded into a debate between direct and indirect approaches. Intuitively, direct approaches seem the most informative in this regard, as subjects must communicate their own experiences^14^. However, as subjective reports have demonstrable limits (e.g. lack of insight into personal bias, memory problems etc.), some scientists refrain from their use and insist on the use of objective measures only^15^.

In the context of research on the bodily self, which often involves inducing illusory ownership over fake bodies/body parts (body illusions), subjective measures typically take the form of questionnaires, which include made-up of statements, such as “I felt as if the rubber hand was my hand”, to which participants respond on a Likert scale^16^. “Objective” measures of these illusions include proprioceptive (hand) drift and skin conductance responses to threats applied to the embodied hands/bodies^17,18^. Within this Collection, rubber hand illusion (RHI) studies used both subjective (embodiment questionnaire) and objective (including proprioceptive drift) measures to examine the effect of the RHI on peripersonal space and perceived position of the body midline^19^. Although global findings across the different measures demonstrated similar effects for both of these studies, dissociations were also apparent between the different types of measures. This suggests that different objective and subjective measures are vulnerable to different biases or may be capturing different aspects or levels of bodily self-representation.

Even in the pursuit of a gold-standard objective measure, when considering the intrinsic link between subjectivity and the bodily self, there seems no way around using subjective reports^20^. In order to arrive at any “stand-alone”’ objective method, one must have “calibrated it” with something else in order to know that this particular behaviour can be considered a measure of—e.g. body ownership—and not something else. This would typically involve *reliably* associating a subjective report with a particular behaviour—a process by which one would “import” all the weaknesses related to subjective reports that one tried to avoid in the first place. Accordingly, we should learn to live with the inclusion of subjective measures in study design, and do our best to develop them to overcome their methodological problems.

Another central question is how to think of the bodily correlates of a self in this basic sense. Modern scientific thinking normally dictates that we cannot conceive of a mental phenomenon that does not have a specific neural (physical) counterpart. Nevertheless, with the basic understanding of the self mentioned above, subjectivity is an intrinsic aspect of all mental functions: Our perceptions, thoughts, emotions, and decisions are all our own. For this reason, it may seem unrealistic to identify one neural region or process that is specific for subjectivity so that it turns it “on” and “off”—without turning everything mental “on” and “off” in parallel. In turn, this may force us to rethink how we conceive of mind–body relations. The recent surge in publications on interoception—the brain’s processing of internal bodily signals—highlights the importance of the ever-present sensory input from the body to the brain and how it may be a crucial component enabling subjectivity. Damasio^21^ proposed the importance of interoceptive brain areas to self long before recent empirical studies demonstrated links between interoceptive processing and consciousness^22^ and self ^23,24^. In support of this, the paper by Saini and colleagues^25^ within this Collection propose that depersonalisation disorder—a condition in which there is a profound alteration to the experience of self and subjectivity—may arise from a disrupted integration between interoceptive and exteroceptive signals. Many more studies on the links between interoception and self are needed, but experimental studies on interoception have been plagued by the confounds affecting methods to measure interoceptive accuracy. Here, Wallman-Jones et al.^26^ report that vigorous physical activity increases interoceptive accuracy irrespective of attentional focus.

Although questions about how to understand relations between mind, brain, and the rest of the body are fundamental and very difficult to answer theoretically, continued methodological developments will hopefully lead to a growing body of evidence suggesting concrete links between the above. These links are what science has to explain in the coming decades, and they already demonstrate what we have historically overlooked—that the body shapes the mind and vice versa.
